# Transdisciplinary Bioblitz: Rapid biotic and abiotic inventory allows studying environmental changes over 60 years at the Biological Field Station of Paimpont (Brittany, France) and opens new interdisciplinary research opportunities

**DOI:** 10.3897/BDJ.8.e50451

**Published:** 2020-03-27

**Authors:** Annegret Nicolai, Muriel Guernion, Sarah Guillocheau, Kevin Hoeffner, Pascaline Le Gouar, Nelly Ménard, Christophe Piscart, Dominique Vallet, Morgane E. T. Hervé, Elora Benezeth, Hughes Chedanne, Jérémie Blémus, Philippe Vernon, Daniel Cylly, Hoël Hotte, Grégoire Loïs, Barbara Mai, Grégoire Perez, Tiphaine Ouisse, Cécile Monard, Claudia Wiegand, Jean-Pierre Caudal, Alain Butet, Maxime Dahirel, Lou Barbe, Manon Balbi, Valérie Briand, Myriam Bormans, Maryvonne Charrier, Guillaume Bouger, Vincent Jung, Cécile Le Lann, Alexandrine Pannard, Julien Petillon, Yann Rantier, Dominique Marguerie, Kevin Tougeron, Pierre Devogel, Sébastien Dugravot, Thomas Dubos, Maël Garrin, Mathurin Carnet, Clément Gouraud, Audrey Chambet, Joël Esnault, Maxime Poupelin, Erik Welk, Astrid Bütof, Glenn F. Dubois, Guillaume Humbert, Odile Marie-Réau, Olivier Norvez, Gaëlle Richard, Benoît Froger, Céline Rochais, Martin Potthoff, Khaoula Ayati, Alain Bellido, Alain Rissel, Mathieu Santonja, Jacques-Olivier Farcy, Eric Collias, Lina Sene, Daniel Cluzeau, Régis Supper

**Affiliations:** 1 Université Rennes 1, Station Biologique de Paimpont, UMR-CNRS 6553 EcoBio/OSUR, Paimpont, France Université Rennes 1, Station Biologique de Paimpont, UMR-CNRS 6553 EcoBio/OSUR Paimpont France; 2 MNHN, UMR 7204 CESCO, Paris, France MNHN, UMR 7204 CESCO Paris France; 3 unaffiliated, Rennes, France unaffiliated Rennes France; 4 Université Rennes 1, UMR-CNRS 6553 EcoBio/OSUR, Rennes, France Université Rennes 1, UMR-CNRS 6553 EcoBio/OSUR Rennes France; 5 Université Rennes 1, Observatoire des Sciences de l'Univers de Rennes (OSUR), UMS 3343, Rennes, France Université Rennes 1, Observatoire des Sciences de l'Univers de Rennes (OSUR), UMS 3343 Rennes France; 6 Université Rennes 1, EA 7462 G-TUBE, Rennes, France Université Rennes 1, EA 7462 G-TUBE Rennes France; 7 Groupe Mammalogique de Bretagne, Redon, France Groupe Mammalogique de Bretagne Redon France; 8 GRETIA, Rennes, France GRETIA Rennes France; 9 Université Rennes 1, Collections de botanique et herbiers, Rennes, France Université Rennes 1, Collections de botanique et herbiers Rennes France; 10 Conservatoire Botanique National de Brest, Brest, France Conservatoire Botanique National de Brest Brest France; 11 CPN les p'tites natures de Brocéliande, Paimpont, France CPN les p'tites natures de Brocéliande Paimpont France; 12 Martin-Luther-Universität Halle-Wittenberg, Halle, Germany Martin-Luther-Universität Halle-Wittenberg Halle Germany; 13 unaffiliated, Halle, Germany unaffiliated Halle Germany; 14 Yandra, Concoret, France Yandra Concoret France; 15 Université Pierre et Marie Curie, Paris, France Université Pierre et Marie Curie Paris France; 16 CNER, Rennes, France CNER Rennes France; 17 Agence Française de la Biodiversité, Cesson-Sévigné, France Agence Française de la Biodiversité Cesson-Sévigné France; 18 Université Rennes 1, Collection Zoologie, Rennes, France Université Rennes 1, Collection Zoologie Rennes France; 19 Bretagne Vivante, Brest, France Bretagne Vivante Brest France; 20 Université Rennes 1, Station Biologique de Paimpont, UMR 6552 EthoS, Paimpont, France Université Rennes 1, Station Biologique de Paimpont, UMR 6552 EthoS Paimpont France; 21 Universität Göttingen, CBL, Göttingen, Germany Universität Göttingen, CBL Göttingen Germany; 22 Faculty of Science of Bizerte, Zarzuna, Tunisia Faculty of Science of Bizerte Zarzuna Tunisia; 23 Encyclopédie de Brocéliande, Paimpont, France Encyclopédie de Brocéliande Paimpont France; 24 Aix Marseille Univ, Avignon Université, CNRS, IRD, IMBE, Marseille, France Aix Marseille Univ, Avignon Université, CNRS, IRD, IMBE Marseille France; 25 Université Rennes 1, DSI, Rennes, France Université Rennes 1, DSI Rennes France; 26 Université Rennes 1, Station Biologique de Paimpont, Paimpont, France Université Rennes 1, Station Biologique de Paimpont Paimpont France

**Keywords:** ATBI, citizen science, terrestrial, land use, aquatic, historical biodiversity data, physico-chemical parameters, photographical landscape observation, soil biota, plant communities, multi-habitat cartography, long-term survey, multi-trophic sampling

## Abstract

**Background:**

The Biological Field Station of Paimpont (Station Biologique de Paimpont, SBP), owned by the University of Rennes and located in the Brocéliande Forest of Brittany (France), has been hosting student scientific research and field trips during the last 60 years. The study area of the SBP is a landscape mosaic of 17 ha composed of gorse moors, forests, prairies, ponds and creeks. Land use has evolved over time. Historical surveys by students and researchers focused on insects and birds. With this study, we aimed to increase the range of taxa observations, document changes in species composition and landscape and provide a basis for interdisciplinary research perspectives. We gathered historical data, implemented an all-taxon biodiversity inventory (ATBI) in different habitats of the SBP study area, measured abiotic factors in the air, water and soil and performed a photographical landscape observation during the BioBlitz held in July 2017.

**New information:**

During the 24 h BioBlitz, organised by the SBP and the EcoBio lab from the University of Rennes and the French National Center of Scientific Research (CNRS), different habitats were individually sampled. Seventy-seven experts, accompanied by 120 citizens and 12 young people participating in the European Volunteer Service, observed, identified and databased 660 species covering 5 kingdoms, 8 phyla, 21 classes, 90 orders and 247 families. In total, there were 1819 occurrences including records identified to higher taxon ranks, thereby adding one more kingdom and four more phyla. Historical data collection resulted in 1176 species and 4270 occurrences databased. We also recorded 13 climatic parameters, 10 soil parameters and 18 water parameters during the BioBlitz. Current habitats were mapped and socio-ecological landscape changes were assessed with a diachronic approach using 32 historical photographs and historical maps. The coupling of historical biodiversity data with new biotic and abiotic data and a photographic comparison of landscape changes allows an integrative understanding of how the SBP changed from agriculturally-used land to a managed natural area within the last 60 years. Hence, this BioBlitz represents an important holistic sampling of biodiversity for studies on trophic webs or on trophic interactions or on very diverse, but connected, habitats. The integration of social, biotic and abiotic data opens innovative research opportunities on the evolution of socio-ecosystems and landscapes.

## Introduction

Biodiversity is severely threatened on a global scale, due to extensive alteration of habitats, over-exploitation, introduction of exotic species, environmental pollution and climate change, subsequently affecting humanity (Pimm et al. 2014). Inventories of biodiversity at various taxonomic levels and spatio-temporal scales were deemed necessary by the Science ­Policy Platform on Biodiversity and Ecosystem Services (Díaz et al. 2015) to reach the Convention on Biological Diversity’s Aichi Targets of the Strategic Plan for Biodiversity 2011–2020 (https://www.cbd.int/sp/targets). Knowledge of past and current biodiversity is thus needed for management decisions from local to global scales. Long-term biodiversity observations, coupled with environmental parameters estimations, are necessary tools to assess ecological changes and ecosystem trends in the face of numerous threats to biodiversity.

Specifically, participative biodiversity inventories address a component of Aichi target 19, that is, to improve and disseminate biodiversity knowledge. Participative biodiversity inventories through citizen science (scientific research involving volunteers for collecting and processing data, Silvertown 2009) contribute worldwide to 55% of species records in the database of the Global Biodiversity Information Facility (GBIF, www.gbif.org, Chandler et al. 2017). While more than half of all citizen science projects in Europe publish their data through GBIF, less than 1% of citizen science data are provided by France (Chandler et al. 2017). There is a great need for participative biodiversity assessments in France, for example, BioBlitzes as citizen science events (Silvertown 2009) and historical citizen science (Clavero et al. 2017), to improve biodiversity knowledge and to increase the awareness and therewith the valuation of species in a given area. During a BioBlitz, experts and the general public intensively survey and map as many species as they can in one location over a given, short time, usually 1-2 days (Lundmark 2003) thereby providing essential biodiversity variables (Pereira et al. 2013), such as taxonomic diversity data at local scales (Chandler et al. 2017). Current citizen science data, combined with historical data, allow assessment of biodiversity change (Clavero et al. 2017). Greater biodiversity knowledge and appropriation of this knowledge by the wider public ensures a strong pressure on public policies to perform efficient conservation actions.

Located in a rural, agricultural region in western France, the study area around the Biological Field Station of Paimpont (Station Biologique de Paimpont, SBP) offers an excellent outdoor laboratory for studies of spatio-temporal ecosystem changes. This station is attached to the University of Rennes and hosts scientists and their collaborators from many different disciplines and countries. The SBP is particularly attractive because of its location in the socio-ecosystem of the Brocéliande Forest which is composed of arable fields with remains of a historical hedgerow system, as well as of mixed wood forest (deciduous/hardwood and softwood), moors, meadows, wetlands, many ponds and creeks.

The Brocéliande Forest has a rich historical and natural heritage. Studies led by researchers from the University of Rennes showed that human activity has influenced the landscape structure and vegetation composition since the Holocene (Marguerie 1992, Marguerie 2009, David 2014, Gaudin 2014, Oillic 2011). Paleo-environmental analyses and data of 258 historical documents showed 13 zones of different pollen composition during 13,000 years of Brocéliande Forest’s history (Briard et al. 1989, David 2014, Marguerie 1992). The historical dynamic of the Brocéliande socio-ecosystem was mainly driven by metallurgical activity (until the end of the 19^th^ century), silviculture and agriculture. Agricultural activities included pastoralism in moors and cereal crops (e.g. buckwheat) on arable land. Currently, silviculture and dairy farms (using temporary prairies and cereal cultures) prevail in Brocéliande Forest.

The SBP has the advantage of being close to stakeholders and territorial decision-makers, and allows recording the changes in an area representative of many other areas currently suffering from major anthropogenic disturbances. Ecosystem characteristics and changes are perceived differently in different socio-economic groups and can lead to contrasting opinions and decisions regarding landscape management (Scott 2006, Woods 2003). Therefore, local approaches with direct knowledge acquisition and transmission are important. In these approaches, photography showing landscape particularities from different viewpoints and from different periods is a widely-used tool to understand factors that influence landscape perception. Hence, landscape changes documented by photographs are an asset of landscape ecology, allowing development of socioecological research approaches while contributing to public outreach (e.g. photo exhibitions, Paradis and Lelli 2010).

## General description

### Purpose

The objectives of this study are (i) to increase the range of taxa observations from the last 60 years in the study area of the SBP, (ii) to provide a basis for analysing spatio-temporal changes in biodiversity and landscape in the SBP study area as a socio-ecosystem model from Brittany, (iii) to launch future interdisciplinary research perspectives in the SBP study area and (iv) to increase awareness and valuation of biodiversity by the public. During the Bioblitz (Lundmark 2003) held in July 2017, we gathered historical data and implemented an all-taxon biodiversity inventory (ATBI, Janzen and Hallwachs 1994) in the different habitats of the SBP study area. These observations were supplemented by measurements of physico-chemical parameters in the air, soil and water, as well as by diachronic photographical landscape observations and filming/interviewing of human-nature relationships in order to provide a socio-ecological approach for future research at the SBP.

## Project description

### Title

The BioBlitz held in 2017 at the SBP was organised in two different phases: (1) biodiversity data collation from research reports and education activities up to 60 years prior to the BioBlitz event and (2) species survey and abiotic parameters during 24 h on 18-19 July 2017, called the “SBP BioBlitz” (https://stationbioblitz.sciencesconf.org).

### Personnel

In total, 77 experts from 14 institutions, mainly from the EcoBio lab of the University of Rennes, but also from NGOs and nature conservatories (Suppl. material 1) were involved in the BioBlitz. The gathering of 60 years’ biodiversity data involved 6 experts from the EcoBio lab of the University of Rennes and the NGOs “Encyclopédie de Brocéliande”, “Groupe Mammalogique de Bretagne” and “GRETIA”. First, an inventory of study reports, PhD theses and survey data sheets was implemented. Historical data were mainly produced by the University of Rennes (France), the University Paris-Sud (France) and the University Liège (Belgium). After extraction, the data were verified, geo-localised and gathered in a common file under an occurrence format. Additionally, landscape photographs older than 60 years were provided by the NGO “Encyclopédie de Brocéliande” which collected old photographs and postcards from personal archives of inhabitants, territorial archives and libraries. During the BioBlitz, 120 citizens helped the experts to implement inventories; either they accompanied experts or explored a zone on their own to collect samples or photograph specimens. The non-expert participants were also invited to sort samples, label collected material and start taxon identifications. They were briefly trained for each task. Species identifications of non-experts were systematically verified by experts. All participants could enter records in a data template file, either their proper records or the data provided by field sheets or by people identifying species in the lab. The 12 European volunteers were involved in logistical organisation, habitat delimitation and communication.

### Study area description

**Figure 1.**
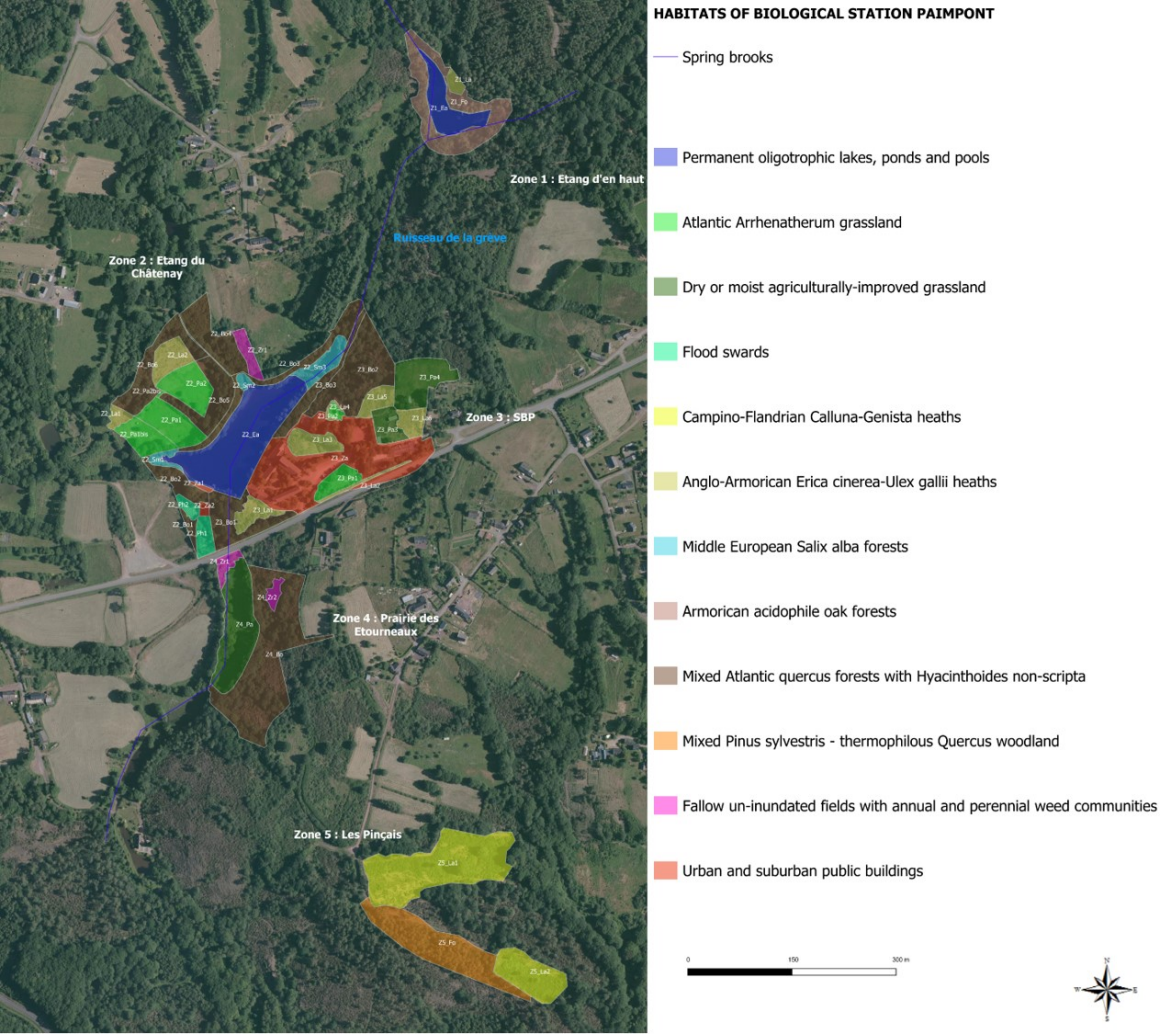
Study area of the Biological Field Station of Paimpont and habitat codes used during the BioBlitz 2017 for different sites.

The mission of the SBP is to preserve the ecological integrity of the area, while providing opportunities for scientific research, student field work and public education. Located on sedimentary rock (shale and sandstone), the climate is temperate oceanic with local specificities due to the topography (hilly landscape with the highest peak at 258 m) and mixed land-cover. The SBP study area covers a diversity of habitats including ponds and creeks, swamps, cliffs, meadows, moors, shrub thickets and forest (Fig. 1, Suppl. material 2). Due to this diversity of habitats, SBP has been the site of a variety of innovative research studies, including the ecology of mammals, birds and insects; fire ecology; phytosociology of bogs, cliffs and forest; landscape ecology; and soil biology. Moreover, the zones “Etang d’en haut” and “Les Pinçais” (Z1 and Z5, respectively, Fig. 1) are zones of ecological, faunistic and floristic interest (ZNIEFF) belonging to the national inventory of natural heritage sites.

The study area of the SBP is composed by a diverse set of land parcels of different historical use in agriculture and local activity, purchased by the University of Rennes. In former times, today’s prairies were arable land, moor was grazed or regularly thinned, hedges represented firewood resource (i.e. trees were pruned), the pond served as a mill and fishing, pastoralism and hunting were practised in woodland. Current management of the study area includes rotating horse grazing on meadows and moor, mechanical thinning of creek and pond banks as well as of hedges and extensive mowing of grassland around the SBP buildings. A trail system across the study area is maintained through mowing, mechanical thinning and installation of board walks. Public access is restricted spatially to the trail around the pond and the area around the buildings of SBP and to public outreach events, such as the Science Festival, Open House and citizen science events, such as the BioBlitz.

Prior to the BioBlitz, in July 2017, the habitat zones were characterised, delimited and mapped. During the BioBlitz 2017, terrestrial sampling was performed in 42 different sites, aquatic sampling was performed in 4 different sites and the habitat map was adjusted and corrected after the BioBlitz using current biotic and abiotic observations (Fig. 1, Suppl. material 2).

### Design description

The BioBlitz, held in 2017 at the SBP, aimed to implement a species inventory including records from 60 years of SBP research and education activities, to collect abiotic data from terrestrial and aquatic habitats and to integrate observations of landscape changes in relation to human activities that occurred during the last 100 years. Collection efforts focused on taxa historically recorded in all habitats of the 17 ha study area at the SBP (Fig. 1, Suppl. material 2). One broom moor site (Z5 La2, Fig. 1) could not be included in the inventory because horses owned by the SBP were kept there. All samplings were performed between 2 pm and ­8 pm (July 18th), 10 pm and 9 am (July 18th-19th) and 10 am­ and 2pm (July 19th). The pollinator observation was extended to July 20th because of unfavourable weather conditions on July 19th. Collembola sampling took place two weeks before the BioBlitz, because the expert could not participate on the chosen BioBlitz dates. Other data, opportunistically collected by participating experts during the year 2017, were also integrated into the dataset. Records were mostly visual and non-invasive observations, because the SBP puts a high priority on biodiversity conservation and protection. However, some biodiversity components were collected to allow identification at the lowest possible taxonomic rank. Sorting of collected samples and species identification mainly occurred on July 20th and 21th. Some identifications of species were conducted by experts up to three months after the BioBlitz. As a taxonomic reference, TaxRef V10.0 (Gargominy et al. 2016) was used for animals and Flora Europaea for plants (http://eunis.eea.europa.eu/references/1780/species).

## Sampling methods

### Sampling description

During this transdisciplinary BioBlitz, species presence was recorded in each habitat and some protocols also measured abundance or biomass. Abiotic parameters were measured in aquatic habitats, in the soil and in the air using the meteorological station on site. Socio-ecological observations included filming and photographing. The methods are described below:

**Birds**

Birds have been observed between 2 pm and 7 pm (18 July) and 7 am–12 pm + 1 pm-2 pm (19 July) using opportunistic prospecting on a total length of 6200 m in all habitat zones of the SBP study area (Fig. 1). They were visually and audially identified. Walking speed was 0.5 km.h^-1^ and every 10 minutes, there was a stop of one minute for audio detection.

**Mammals**

Mammals inventory was carried out in all habitats of the SBP study area (Fig. 1). Species records are direct observations of animals or signs of recent presence (fresh dung, fresh track, fresh food scraps, deer smear, dens, other specific traces) which were collected along paths of least cost. In addition, five camera traps were installed in two sites, Z4_Bo, Z5_Fo and Z1_La (Suppl. material 3), two days before the BioBlitz to minimise disturbance by human smell.

Small mammal inventory was done by using live trapping (Perez et al. 2017). Thirteen trap lines were set in different habitats of the SBP including woodland, meadows, moors and wetlands (Suppl. material 3). Each trap line featured 34 INRA traps with baited dormitory boxes and were set on 18th of July and checked in the morning the next day. Trapping effort was equivalent to 442 trap nights.

Bats were trapped using mist nets in one site, Z2_Bo4 (Suppl. material 3) and recorded based on their echo-location signals captured with D-1000X Bat Detector (Pettersson Elektronik AB, Uppsala, Sweden).

**Amphibians and Reptiles**

Amphibians and reptiles have been observed using opportunistic prospecting in all habitat zones of the SBP study area (Fig. 1). This method includes searching under decaying logs and rocks, prospecting nesting sites and using nets in ponds.

**Plants, lichen and mosses**

Vascular plants, lichens and mosses were surveyed using opportunistic prospecting of all habitat zones of the SBP study area (Fig. 1). For identification of species, the Flore des Abbayes (DesAbbayes et al. 2012), Flora Gallica (Tison and Foucault 2014), Flore Forestière Française (Rameau et al. 1999) and Rothmaler (Jäger et al. 2017) were used.

**Soil biota**

Soil and litter dwelling invertebrates were sampled at 37 points (Suppl. material 3) evenly distributed over the SBP study area. At each point, three sampling methods were used on a 1 m^2^ plot: (i) pitfall traps (Spence and Niemelä 2012), (ii) board traps (Boag 1982) and (iii) soil extraction (0-10 cm deep, 5 cm diameter), followed by the Tullgren funnel method (Berlese 1905) over a 7-day period. Additionally, a 10 min visual search was performed 10 m around the plot. Barber traps were made of plastic pots (10 cm deep, 12 cm diameter), filled with salted water (30% v/v) and detergent (3% v/v) and buried to the rim into the soil, five days before the BioBlitz. In sites where natural wood traps, such as decaying logs, were available, they were included in the survey. Otherwise, board traps were made of poplar wood (30-40 x 50-60 x 2-3 cm) and placed flat on the ground, five days before the BioBlitz beside the pitfall traps. Individuals, that could not be identified in the field, were stored in 95% ethanol for later identification using binocular loupes. Collembolans were identified to species rank (Gisin 1960, Hopkins 1997).

Bacteria were quantified using qPCR (Heid et al. 1996). Bioluminescent organisms were randomly searched for at night in the forested areas around the pond “Etang du Châtenay” (Z2_Ea, Fig. 1).

**Insects (above-ground)**

Invertebrates in trees and shrubs were surveyed using a beating sheet with vegetation shuffling and a sweep net in each habitat type (Fig. 1). Pollination insects were photographed individually during 20 min on individual flowering plants following the SPIPOLL protocol (Suivi Photographique des insects pollinisateurs - photographic survey of pollinators, www.spipoll.fr). The plant was identified and recorded as host for each pollinator.

For nocturnal insects, night sheets were installed on the dyke of the pond “Etang du Châtenay” (Z2-Za1, Fig. 1) and in the prairie (Z2-Pa1, Fig. 1) and sampling was implemented between 11 pm and 3 am (18-19 July). Additionally, a light trap was installed in the prairie near the weather station (Z3-Pa2, Fig. 1) between 10 pm and 5 pm (18-19 July).

**Aquatic organisms**

Aquatic meso-invertebrates were qualitatively sampled in the different freshwater habitats in four sampling sites (two in the stream upstream and downstream of the pond Etang du Châtenay and in the two ponds) using the kick-sampling method with a 0.25 mm mesh hand net. The net contents were placed into a shallow white tray with enough water to allow invertebrates to swim or crawl. Alive invertebrates were sorted directly in the field and preserved in 96° alcohol until their identification under a stereomicroscope SZX16 (Olympus).

For plankton analysis, water samples were taken in sub-surface (-10 to -20 cm) in the centre of the two water bodies (pond “Etang du Châtenay” Z2-Ea, pond “Etang d’en Haut” Z1-Ea, Fig. 1). In order to estimate the total phytoplankton biomass, the concentration of chlorophyll "a" was measured in situ, using a probe Ocean Seven 316+ probe (Idronaut, Brugherio, Italy), equipped with a Trilux multiparameter algae sensor (Chelsea, Surrey, United Kingdom). Then in the laboratory, the chlorophyll "a" measurement was used, according to the method of Lorenzen (2003). To determine the composition of the plankton community, 250 ml of water was fixed with lugol iodine acid solution (Throndsen and Sournia 1978) and stored at 4°C, until counting with an optical microscope after filtering with a bolting cloth of 60 µm mesh. A fresh sample was also observed within 24 hours of collection to identify the species present.

Fish were caught with a baited crawfish trap and released after identification.

**Landscape photography**

**Figure 2.**
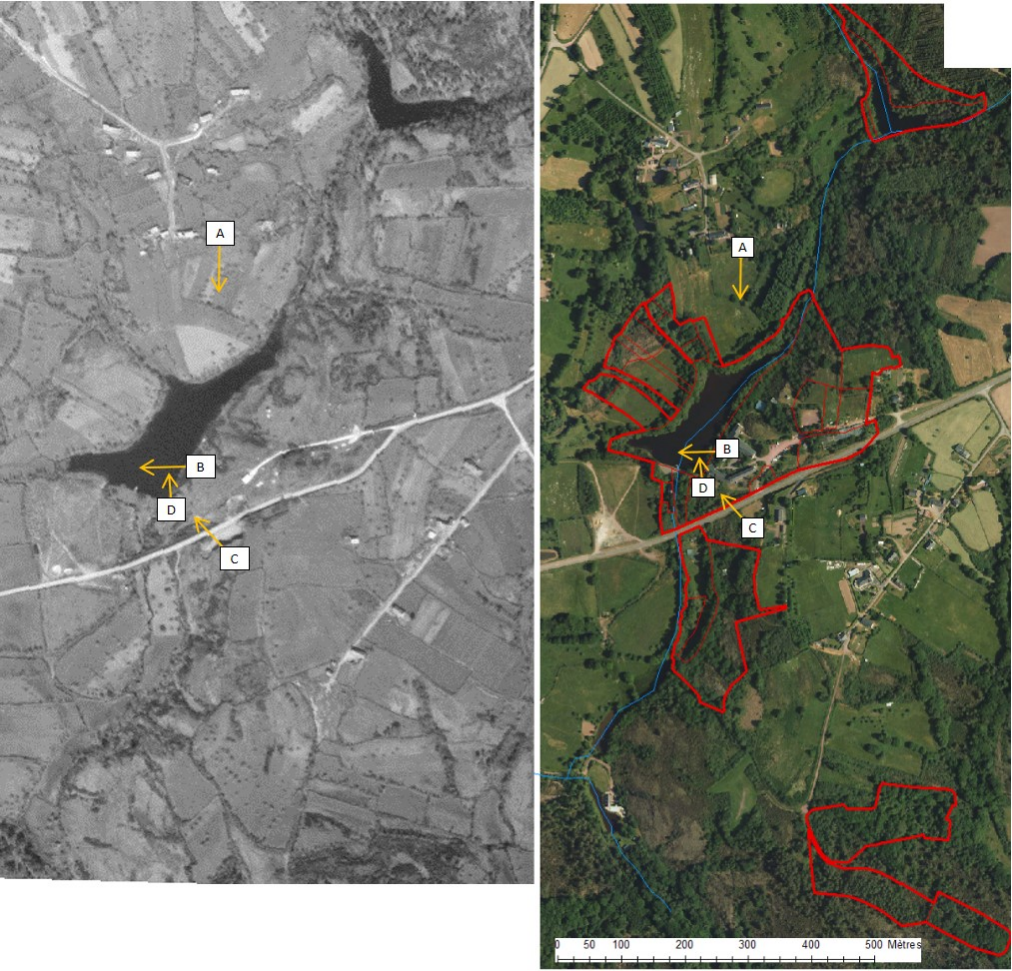
Aerial photos of 1950 (left) and 2013 (right) with the study area of the Biological Field Station of Paimpont and localisation of landscape photographs (A: N48°00'22", W2°13'48"; B: N48°00'13", W2°13'47"23; C: N48°00'9", W2°13'46"20; D: N48°00'11", W2°13'48") with arrows for viewing direction.

Out of a collection of historical photographs ranging from around 1900 to 2016, databased by the NGO “Encyclopédie de Brocéliande”, 32 viewpoints were chosen to reconduct. The diachronic observations were described and grouped into different categories that could best describe landscape changes within the study area of the SBP over time. The viewpoints of the four most representative photographs were mapped on two aerial photographs, one from 1950, prior to the construction of the SBP and one as of 2013 (Fig. 2).

**Ecosemiotic interviews**

Filmed interviews were implemented with BioBlitz participants to explore how humans perceive and translate or are affected by the exchange of signs with and amongst non-human organisms within the biosphere. In the search for a “systemic wisdom” (Bateson 1972), the film material presents human-non-human relationships beyond categorisations reified by our symbolic language.

**Physico-chemical parameters**

Historical data of abiotic parameters are available in various documents, but are not accessed yet. The weather station at the SBP recorded several parameters going back to 1958. These data can be communicated upon request. During the BioBlitz, soil, water and climatic parameters have been recorded using the following methods:

*Soil parameters*

Top soil sampling was performed on three different sites within the study area of the SBP: a gorse moor (Z2-La2), an oak forest (Z2-Bo5) and a meadow (Z2-Pa1, Fig. 1). One sample per site was sent to LABOCEA Comburg for standardised analyses (NF ISO or NFX norms) of total nitrogen and C/N (NF ISO 13878), granulometry (NFX 31107), organic matter (NF ISO 10694/14235), residual humidity (NF ISO 11465), cation exchange capacity (NF ISO 23470) and pH. In situ CO_2_ and volatile organic compounds (VOCs) emissions from soil in these three sites were measured four times during the BioBlitz (on the 18th afternoon, twice during the night from the 18th to the 19th and once on the 19th July morning) using dynamic gas sampling in canisters and later analysis on a micro gas chromatography (µGC) and proton transfer reaction mass spectrometer (PTR-MS), respectively (Potard et al. 2017).

*Water parameters*

Aquatic parameters have been measured at four sites, one site per pond and one site per creek downstream of each pond (Z1-Ea, Z2-Ea, Fig. 1) and at the dyke of pond “Etang du Châtenay” (Z2-Ea, Fig. 1), using the Ocean Seven 316+ probe (Idronaut, Brugherio, Italy), equipped with a subaquatic LI-190R Quantum sensor (LI-COR, Lincoln, USA) and a Trilux multiparameter algae sensor (Chelsea, Surrey, United Kingdom). In each pond, two profiles in three sites were obtained (6 profiles in total), except at the dyke and in the creeks where only stationary measurement was possible. Additionally, multiparametric water quality probes 6600-V2 and 6920 (YSI, Yellow Springs, USA) were used in the creeks.

In order to measure the concentrations of nutrients, a water sample was taken in the sub-surface (-10 to -20 cm) at the centre of the ponds. A volume of water was then filtered through Whatman GF/F filter (0.7 μm) for dissolved nutrients (nitrate and phosphate) and all samples were stored at -20°C. Measurements of nutrient concentrations were performed by colourimetric methods (Henriksen and Selmer-Olsen, 1970, Murphy and Riley, 1962) using a Bran and Luebbe Autoanalyzer 3 (Axflow, Norderstedt, Germany). Nitrate was measured after reduction to nitrite on a cadmium-copper column (Henriksen and Selmer-Olsen 1970). Phosphate was measured following the method of Murphy and Riley (1962). Total phosphorus (TP) and total nitrogen (TN) concentrations were analysed after a hot alkaline persulphate digestion (Aminot and Chaussepied 1983). Total dissolved organic carbon (DOC) was measured by chemical mineralisation using a carbon analyser (OI – Analytical).

*Climatic parameters*

Climatic parameters were obtained from the weather station of the SBP, located in Z3-Pa2 (Fig. 1). Automated measurements were performed every 2 seconds.

## Geographic coverage

### Description

The Biological Field Station of Paimpont (Station Biologique de Paimpont, SBP, https://station-biologique-paimpont.univ-rennes1.fr) in the Brocéliande Forest in continental Brittany, North-West France (48.00 N, -2.22 W) is surrounded by a study area of 17 ha owned by the University Rennes.

### Coordinates

47.993 and 48.011 Latitude; -2.2359 and -2.2019 Longitude.

## Taxonomic coverage

### Description

**Figure 3.**
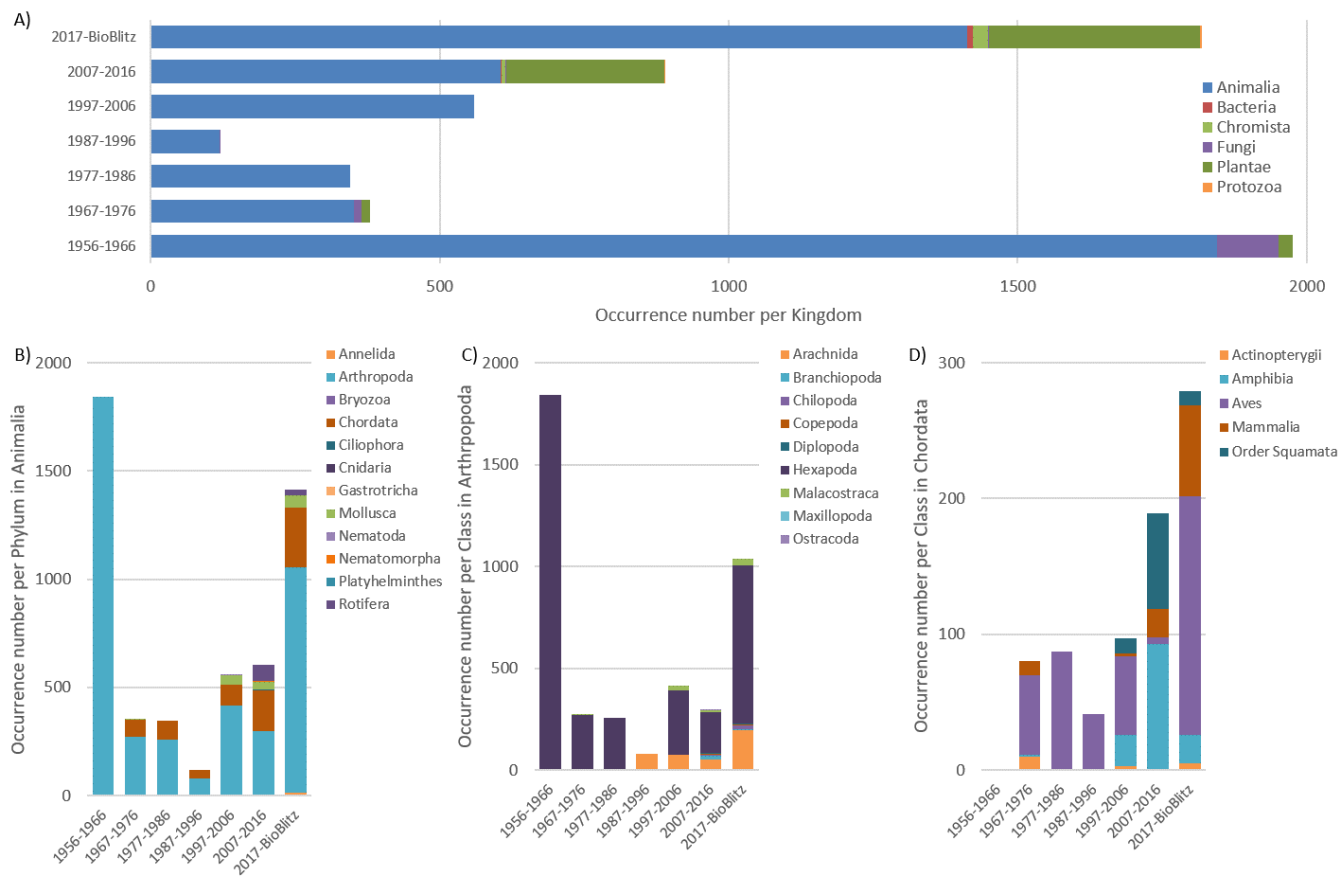
Number of occurrences per Kingdom (taxon rank) (A), per animal Phylum (B), per arthropod Class (C) and per vertebrate Class (D), within 10 years intervals since 1956, as well as within 2017 including the BioBlitz on July 18-20 at the Biological Field Station Paimpont, France. Note that the first interval is 11 years.

During 60 years prior to the Bioblitz, 1176 species (Table 1) with 4270 occurrences were recorded (Fig. 3A). During the Bioblitz, 660 species, 5 kingdoms (following Ruggiero et al. 2015), 8 phyla, 21 classes, 90 orders and 247 families were covered, of which many species were already observed during the last 60 years; most occurrences were from the 1950s (Fig. 3A). However, 343 species were added to the historical list of species during the BioBlitz (Table 1), for instance, twelve new Coccinelidae insects were added. In total, there are 6089 occurrences including records identified to higher taxon ranks from 6 kingdoms and 12 phyla (Fig. 3 A, B). The most diverse groups were plants as well as arthropods, followed by vertebrates in the phylum Animalia (Fig. 3A, B). For arthropods, mainly insects were observed historically, while during the BioBlitz, eight other orders were additionally observed, mostly arachnids (Fig. 3C). In the vertebrate class, birds were the most often recorded historically, while amphibians and mammals received attention in the last decade prior to the BioBlitz and during the BioBlitz (Fig. 3D). The addition of 52 plant species, recorded during the BioBlitz (Table 1) to the botanical inventory at the SBP and the cartographical landscape observations (Fig. 1), led to an improved cartography of habitats (Fig. 1).

In total, our dataset includes 17 rare species ranging from near threatened to critically endangered on French, European or IUCN red lists (Table 1). Plants and arthropods comprise most red-listed species (Table 1). One arthropod species, found during the BioBlitz, was first recorded in Brittany in 2016 (coleoptera *Lebia marginata* Geoffroy in Fourcroy 1785). The moor plant *Euphorbia esula* Linneaus 1753 is rare in Brittany and was only recorded in two central Brittany sites, one of them at the SBP and seven coastal sites. Besides two endemic species (gastropod *Elona quimperiana* Blainville, 1821 and amphibian *Triturus marmoratus* Latreille, 1800), we also recorded 20 introduced species. During the BioBlitz, we also focused on kingdoms of microscopic species, such as Bacteria, Chromista and Protozoa (39 occurrences during the BioBlitz).

### Taxa included

**Table:** 

Rank	Scientific Name	
kingdom	Bacteria	
kingdom	Protozoa	
kingdom	Fungi	
kingdom	Chromista	
kingdom	Animalia	
kingdom	Plantae	

## Temporal coverage

**Data range:** 1956-6-23 – 2017-11-01.

## Usage rights

### Use license

Creative Commons Public Domain Waiver (CC-Zero)

## Data resources

### Data package title

Biological Field Station of Paimpont, University Rennes 1

### Resource link

https://doi.org/10.15468/jgnjsa

### Alternative identifiers

https://www.gbif.org/dataset/4dcfa4f3-7ebe-47d6-bd93-a85315c9f180

### Number of data sets

1

### Data set 1.

#### Data set name

Sbp-BioBlitz2017_60years-BiodiversityData

#### Data format

Darwin Core

#### Number of columns

76

#### 

**Data set 1.** 

Column label	Column description
occurrenceID	unique identifier of the occurrence
basisOfRecord	observation type
eventID	unique identifier of the observation event
eventDate	date of the event
endDayOfYear	number of days corresponding to the eventdate starting on the first day of the year
year	year of the event
month	month of the event
day	day of the event
verbatimEventDate	originally indicated event date
eventRemarks	comments regarding the event
associatedOccurrences	other occurrences with their ID that have been observed in direct association with the occurrence
samplingProtocol	protocol used for the observation of this occurrence (described in the reference or in the data paper)
scientificName	genus, specific epithet and scientific name authorship with year of the occurrence
organismQuantityType	measurement of the observed organism quantity
higherClassification	kingdom, phylum, class, order, family, genus, specific epithet, infraspecific epithet of the occurrence
Kingdom	kingdom of the occurrence
phylum	phylum of the occurrence
class	class of the occurrence
order	order of the occurrence
family	family of the occurrence
genus	genus of the occurrence
specificEpithet	species name of the occurrence
infraspecificEpithet	subspecies name of the occurrence
taxonRank	lowest taxon rank of identification of the occurrence
scientificNameAuthorship	author of the scientific name with year for the occurrence
vernacularName	common name in English and French of the occurrence
identifiedBy	name of the person that has identified the occurrence
dateIdentified	date de l'identification de l'occurrence
nomenclaturalCode	national nomenclature used for the taxonomy of the occurrence
taxonID	reference for the taxon of the occurrence
decimalLatitude	latitude in decimal degrees for the occurrence location
decimalLongitude	longiitude in decimal degrees for the occurrence location
verbatimLatitude	originally indicated latitude
verbatimLongitude	originally indicated longitude
coordinateUncertaintyInMetres	precision radius of the location of the occurrence
verbatimCoordinates	originally indicated coordinates
verbatimCoordinateSystem	originally indicated coordinate system
geodeticDatum	geodetic datum of the coordinate system for the occurrence
georeferencedBy	name of the person that has georeferenced the location of the occurrence
georeferencedDate	date of georeferencing
georeferenceRemarks	method used for georeferencing: either the centroid of each habitat zone/whole SBP property or use of a GPS
georeferenceVerificationStatus	verification of the georeferencing
establishmentMeans	state of establishment of the species in the area
higherGeography	Continent, country, province, county, municipality, locality of the occurrence location
continent	continent of the occurrence location
preparation	method of specimen preservation
otherCatalogNumbers	catalogue numbers in collections
country	country of the occurrence location
countryCode	code of the country of occurrence location
stateProvince	province (region) of the occurrence location
county	county (departement) of the occurrence location
municipality	municipality name of the occurrence location
locality	locality description of the occurrence location: SBP and habitat zone (see Fig. 1)
verbatimLocality	originally description of the locality of the occurrence
locationID	identifier of the location corresponding to the habitat grid at the SBP (see Fig. 1)
locationAccordingTo	team whichhas defined the locations and reference map
habitat	habitat description for each occurrence, based on EUNIS habitat classification (with EUNIS habitat code)
type	type of the observation record that was used for the occurrence
previousIdentifications	former identifications
language	language of the record for the occurrence
licence	licence of data use
bibliographicCitation	citation of the occurrence: either the bibliographic source of the occurrence or the data paper
institutionID	identifier of the institution publishing the dataset
institutionCode	code of the institution publishing the dataset
datasetName	name of the dataset
datasetID	identifier of the dataset
collectionCode	code for the collections used in this dataset
catalogNumber	number of the occurrence in the dataset
recordNumber	number of the record in the collection corresponding to the occurrence
organismRemarks	Red List status
lifeStage	description of life stage of the occurrence
references	reference of the occurrence if published elsewhere
recordedBy	name of the observer or specimen collector of the occurrence
sex	description of the sex of the occurrence
individualCount	number of individuals in the occurrence
organismQuantity	number of organisms in the occurrence

## Additional information

### Abiotic and socio-ecological data collection

To characterise environmental conditions, we recorded 10 soil parameters (Table 2), 13 climatic parameters (Table 3) and 18 water parameters (Tables 4, 5, 6). Whatever the sampling time, CO_2_ and VOC emissions from the meadow soil were slightly higher than from the two other habitats; however, they were also highly variable (Table 2) depending on the weather conditions (Table 3). Just before the BioBlitz start on 18 July, a thunderstorm noticeably changed the climatic conditions (high precipitation and a momentary drop in temperature, Table 3, Suppl. material 4). In the afternoon of 18 July, the temperature had recovered, while the morning of the 19 July was cloudy and temperature stayed low (Table 3, Suppl. material 4). The ponds are quite shallow, the water is of a brown colour and rotifers have been observed in water samples. There was a slight stratification in both ponds which was reflected in all parameters (Tables 4, 5, 6). Oxygen was saturated at 1.6-2.0 m depth coinciding with a small peak in chlorophyll "a". At the bottom, the oxygen concentrations were almost anoxic. There was only a slight variation in the profiles of the three sites per pond (Suppl. material 5).

**Figure 4.**
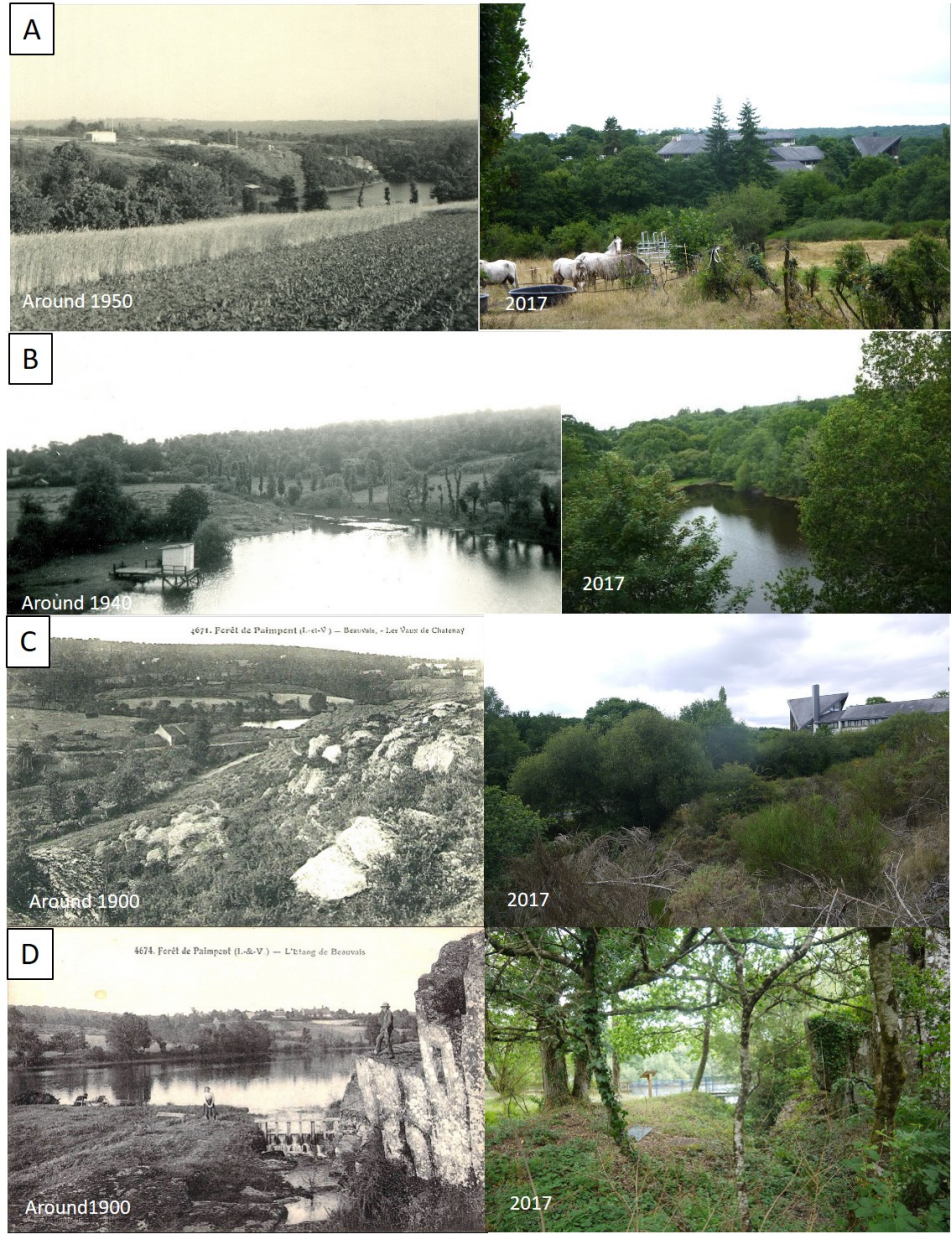
Historical landscape photographs (left side) redone from the same viewpoint (see Fig. 2) during the BioBlitz 2017 (right side) in the study area of the Biological Field Station of Paimpont (SBP). **A.** Pond Etang du Châtenay and cliffs of SBP localisation (habitat zone Z3, Fig. 1); **B.** Pond Etang du Châtenay viewed from the SBP (habitat zone Z2, Fig. 1); **C.** Moor above Prairie des Etourneaux in the foreground (habitat zone Z4, Fig. 1) with the mill, pond Etang du Châtenay and cliff of SBP localisation in the background (habitat zone Z3, Fig. 1); **D.** Dyke of the pond Etang du Châtenay and cliffs (habitat zone Z2, Fig. 1).

There is a considerable number of historical landscape photographs available from Brocéliande Forest. Many of them are postcards made about a hundred years ago when rural life was pictured to reflect a region's identity. Landscape photographs were saved by “Encyclopédie de Brocéliande” (http://broceliande.brecilien.org). Four categories of landscape change observations emerged from 32 diachronic landscape photographs (Fig. 4): (A) construction of SBP and land use/activity changes, (B) evolution of the pond banks, (C) evolution of the moor and (D) appearance of wooded areas. Human activity was and is shaping the landscape around the SBP and is a non-negligible factor when studying biodiversity and human-nature relationship in the socio-ecosystem of Brocéliande Forest. Different ways of envisioning the human-nature relationships emanated from ecosemiotic interviews and filming of participants. Film material for an ecosemiotic analysis has been published by Hypotheses WordPress (https://ecosemiotic.hypotheses.org)

### Outcomes of this transdisciplinary BioBlitz

A substantial number of species was added to the historical record list thanks to the temporally concentrated effort of a high number of participants offering a variety of expertise amongst scientists, naturalists and the curious public (Nicolai et al. 2020). While a BioBlitz event will not provide a complete species inventory, it can add important multi-scaled information within a snapshot (e.g. Telfer et al. 2015). We now have a quantity of outstanding sampling data which integrates species occurrences (from micro- to macro-organisms), biotic and abiotic measurements (VOC, water parameters etc.) and landscape observations (cartography, sociological approach via landscape photography etc.). Moreover, the long-term observations implemented in the SBP study area, generally using standardised methods, allow us to analyse changes in communities, trophic webs and functions in the socio-ecosystem with regards to the evolution of anthropogenic pressure, climate change and alternative management practices over the past 60 years.

This Bioblitz has contributed to an increase in current knowledge on biodiversity in the study area of the SBP. On one hand, many new species, for instance arthropod species, that have been absent from historical data, are actually very common and abundant. The historical lack of data was not due to lack of experts or low search effort, but to incomplete recording. On the other hand, despite the focus on verifying the existing checklist based on historical data, many species could not be found. This shows that generalist insect species dominate the current ecosystems, while specialist insect species are now rare or have disappeared from agro-ecosystems as observed, for example, in butterflies (Nilsson et al. 2008). In total, 80% of insect biomass has declined in Europe over the last 30 years (Hallmann et al. 2017). Birds feeding on insects show the same tendency in France (30% decline over the last 30 years, MTES 2018), indicating cascade effects in trophic webs linked to insecticide use in agriculture (Hallmann et al. 2014). Bird species, detected during the BioBlitz, only represented 52% of the list of the total species established from historical data. Thus, our dataset and the ongoing monitoring at the SBP may contribute to understanding the impact of the agricultural development as forecast by Tilman (2001).

Overall, we have a quantity of outstanding sampling data for arthropod species allowing for diachronic comparison (1956-1966 versus 2017), especially for moths. Within the 2007-2016 time frame, our dataset contains repeated measurements of aquatic micro-organisms, like rotifers and chromista, but also a few records of gastrotricha, bryozoa and cnidaria. The bird sampling that started in the 1970s provides a complete time series documenting the changes in community composition. Besides temporal analyses, our dataset also allows for spatial investigations of biodiversity within the mosaic of habitats; 40% of all occurrences (including historical records) are geo-localised according to the habitat grid of the study area (Fig. 1). Specialist species can be related to a specific habitat (EUNIS classification, Louvel et al. 2013; Suppl. material 2), such as the arachnid *Agyneta rurestris* occurring only in Middle European Salix alba forest (Z2_Sm2, Fig. 1), while more generalist species were recorded in several habitats.

Although the taxon expertise range was quite large amongst the BioBlitz participants, there are still some few under-surveyed groups at the SBP because of the lack of available experts, such as fungi/lichens (only two observations during the BioBlitz compared to 89 species in the historical dataset), bacteria and chromista (only few records identified to lower taxon ranks) and mosses (only historical observations) (Table 1). For microbiota, including fungi and bacteria, molecular technics may be more efficient (e.g. Fierer et al. 2007). Amongst the meso- and macro- biota, that can be visually searched or detected with whole-organisms sampling methods, some species were not found during the BioBlitz, but historically recorded. The reason for missing species is a combination of (i) inappropriate sampling time, (ii) changes in community composition, (iii) local or regional extinctions as explained below:

(i) Some species, recorded in the past, might have some life histories making them undetectable during the dates chosen for the Bioblitz (e.g. invertebrates present as larval instars or eggs). Others show some phenological patterns that would ease sampling in a different season, such as some bird species (spring sampling: territorial songs are produced during breeding season), amphibians (spring sampling: presence in breeding sites) and bats of the genus *Myotis* or *Plecotus* (winter sampling). In the same way, for reptiles (snakes and lizards), their detectability is also largely conditioned by weather factors and the season (not favourable at the BioBlitz date).

(ii) The study area at the SBP progressively re-naturalised due to changes in land use and a decrease in anthropogenic pressure, linked to abandoning agricultural and other human activities (Fig. 2). Our photographic landscape observations show a general increase in woodland and denser vegetation in old-growth moors (Fig. 4). Hence, these vegetation changes within the landscape might be reflected in animal community composition of different habitats as observed in Mediterranean snails (Labaune and Magnin 2002). Open-land species may have been substituted by forest species.

(iii) Several species in the historical data are now considered as endangered or extirpated at regional or national level. Some birds have not been observed in the SBP study area for more than 10 years, such as the Eurasian Wryneck, *Jynx torquilla* and the Grey-headed Woodpecker, *Picus canus*. Both species were nesting in the SBP study area in the seventies and have now been considered as extirpated amongst Brittany’s nesting birds. Some birds, inhabiting agricultural areas with prairies, meadows and fields, such as the Eurasian Skylark, *Alauda arvensis* or the Meadow Pipit, *Anthus pratensis*, have disappeared from the SBP 10 and 20 years ago, respectively, because of negative effects of agricultural practices (intensification in land and pesticide use, Benton et al. 2002).

Moreover, we have comprehensive abiotic data provided along with biodiversity which allows us to study functional diversity. For instance, the climate and soil parameters combined with soil biota and plants allow us to relate soil functions, such as VOC production, to the biotic and abiotic conditions in the ecosystem (Insam and Seewald 2010). In the ponds, we measured the temperature and conductivity gradient. We also found the typical acidic pH for forest watersheds with low photosynthetic activity (oxygen < 100%). Although degradation processes (by microbiota, such as rotifers) dominate over primary production processes (related to low phosphorus and nitrogen availabilities), chlorophyll "a" values showed some algae activity across the whole water column, because light is also available at the bottom. *Gonyostomum semen* (Raphidophyceae) largely dominated the phytoplankton community indicating the high concentration of humic acids (Cronberg et al. 1988).

Our photographic landscape sampling is a dynamic tool that has been proven to capture the evolution of a territory (Mocquet 2017) and to mediate global change impacts at local landscape scale (Bigando 2013). As there are great differences between people's perceptions of the same environment, photographs can serve as a reference for a common dialogue. The chosen viewpoints and angles, as well as the presence of humans or human activity on the photographs, reflect the historical representation of landscape. Agricultural activity dominated in the historical study area of the SBP. Nowadays, most habitats in the study area are naturalised, while some were completely made by artificial means (SBP buildings, dyke). These perceptions conveyed with the photographs can be crossed with other ecological data of the site (biodiversity data, satellite images, maps) to create socio-ecological indicators for the state of the socio-ecosystem that can provide information on policy and local management strategies (Paradis and Lelli 2010).

### Research perspectives at the Biological Field Station of Paimpont

The evolution from agricultural land to a natural area at the SBP is perfect for studying spatio-temporal changes of communities and associated environmental factors, as well as related ecosystem processes. The SBP’s partnerships with local NGOs allows access to documentation on historical and natural heritage, land use changes, evolution of fishing and hunting practices, use of natural resources etc., going back a few centuries (Oillic 2011). The creation of the Photographical Landscape Observatory, the proximity to local stakeholders and rural population allow for integrative interdisciplinary approaches combining historical, sociological, economical and political sciences with ecology. While some habitats of the study area of the SBP were, are and could be further used as experimental natural sites for investigating, for example, ecosystem functions, trophic webs and long-term changes, the SBP is also currently offering quite a large panel of transdisciplinary socio-ecological research opportunities taking advantage of developing specific research questions in local partnerships:

- National Forestry Office (Office National des Forêts): forest management regarding climate change, impact and management of forest fires etc.
- County Council (Conseil Départemental) of Ille-et-Vilaine: habitat management, touristic development in sensitive natural areas (Espace Naturel Sensible, ENS), effective protection of wetlands and bogs etc.
- Regional Centre for Privately-owned Forests (Centre Régional de la Propriété Forestière, CRPF) in charge of the Natura 2000 network at Paimpont: restoration, alternative management practices, evolution under climate change etc.
- Military school for officers of Saint Cyr Coëtquidan owning 5250 ha of land which is closed to the public since 1843 and used for military exercises: the land is adjacent to the SBP study area and has not been explored for biodiversity yet.
- Paleo-environmental study sites in the Brocéliande Forest allow ecological studies on larger time-scales, based on a comprehensive collection of data going back 13,000 years in history (Suppl. material 6).

In perspective, the outstanding outdoor laboratory at the SBP allows development of integrative research approaches, based on the comprehensive data collection implemented in this study. Long-term observations and an intensive transdisciplinary short-term sampling exercise during the BioBlitz 2017 offers a large panel of research opportunities, student education topics and public outreach possibilities, that are orientated towards innovative integrative socio-ecological approaches in landscape, functional and community ecology.

## Supplementary Material

7A672CE3-8A3E-551D-AD6C-9B2B1EA71631 10.3897/BDJ.8.e50451.suppl1 Supplementary material 1 SBP BioBlitz 2017 contributors Data type Meta data Brief description A list of all contributors who took part in the collection and identification of specimens, as well in the manuscript writing. File: oo_372616.txt https://binary.pensoft.net/file/372616 Same as manuscript

719BD165-73FE-5A08-87BE-A02BC35D1D41 10.3897/BDJ.8.e50451.suppl2 Supplementary material 2 Habitat description at the SBP Data type Attribute table of sampling zones Brief description Habitats in different zones based on the EUNIS classification (Louvel et al. 2013) in the study area at the Biological Field Station of Paimpont (SBP) with habitat codes used during the BioBlitz 2017. File: oo_372617.txt https://binary.pensoft.net/file/372617 Same as manuscript

F54A21D5-4B10-506F-A7AC-0C1543690743 10.3897/BDJ.8.e50451.suppl3 Supplementary material 3 Sampling points Data type Track and point map Brief description Sampling design used during the BioBlitz on 18-19 July 2017 in the study area of the Biological Field Station of Paimpont, France. See Figure 1 for habitats in the study area. File: oo_372618.png https://binary.pensoft.net/file/372618 Same as manuscript

DB4123CC-BEB3-57C1-B009-CF3D227A9D7D 10.3897/BDJ.8.e50451.suppl4 Supplementary material 4 Climatic conditions during BioBlitz Data type Graph Brief description Air temperature and precipitation two days before and the two days of the BioBlitz on 18-19 July 2017 at the Biological Field Station of Paimpont, France. File: oo_372620.png https://binary.pensoft.net/file/372620 Same as manuscript

66569176-9E2A-5D3C-A540-E1F42E3975B2 10.3897/BDJ.8.e50451.suppl5 Supplementary material 5 Profiles of abiotic water parameters Data type Graphs Brief description Physico-chemical parameters in the water column in different aquatic sites measured during the BioBlitz 2017 at the Biological Fied Station of Paimpont, France. File: oo_372624.png https://binary.pensoft.net/file/372624 Same as manuscript

152050AC-C00D-5A15-8389-1CD1665AFC3A 10.3897/BDJ.8.e50451.suppl6 Supplementary material 6 Paleobotanical study sites in Brocéliande Forest Data type Meta data for several studies Brief description Metadata table of paleo-environmental study sites in Brocéliande Forest. Marguerie D. (1992) Evolution de la végétation sous l’impact humain en Armorique du Néolithique aux périodes historiques. Travaux du laboratoire d’Anthropologie de Rennes, 40. University of Rennes 1. Oillic J.-C. (2011) Végétation, peuplement, métallurgie en Brocéliande : étude interdisciplinaire de la forêt de Paimpont (Bretagne, France) depuis la fin du Tardiglaciaire. PhD thesis, University of Rennes 1, France. File: oo_372625.txt https://binary.pensoft.net/file/372625 Same as manuscript

## Figures and Tables

**Table 1.** Summary of records identified to species and their status from the study area at the Biological Field Station of Paimpont extracted from historical documents and obtained at the BioBlitz in July 2017. FR – France, EU – Europe, IUCN – global red list of endangered species. Endangered species status following the IUCN classification (IUCN 2012): NT – near threatened, VU – vulnerable, CR – critically endangered.

**Kingdom**	**Taxonomic groups**	**N species recorded**				**N introduced species**	**N species with conservation status**	
		**historically (1956-2016)**	**during the BioBlitz 2017**	**newly from BioBlitz 2017**	**total**		**Endemic**	**Red listed in FR/EU/IUCN**
** Bacteria **			2	2	2			
** Chromista **			3	3	3			
** Fungi **		89	1	1	90			
** Plantae **	Vascular plants	253	152	52	305	10		2 (NT, VU)/2 (NT)/3 (NT)
	Mosses	19			19			
	Algae	0	2	2	2			
** Animalia **	Rotifers	30	11	8	38			
	Bryozoa	1			1			
	Annelida	2	5	5	7			
	Arthropods	629	374	239	868	6		-/-/4 (NT)
	Molluscs	21	28	17	38	1	1	
	Fishes	7	4	2	9			-/-/1 (CR)
	Amphibians	15	9		15		1	-/1 (NT)/-
	Reptiles	9	8		9			
	Birds	75	43	5	80	1		-/-/2 (NT, VU)
	Mammals	26	18	7	33	2		-/-/2 (NT, VU)
**Total**		**1176**	**660**	**343**	**1519**	**20**	**2**	**2/3/12**

**Table 2.** Physico-chemical parameters measured in the soil of three different habitat types during the BioBlitz in 2017 (day: 18 July, night: 18-19 July) in the study area of the Biological Field Station of Paimpont, University Rennes 1. The exact locations were 48.00486N, -2.231713W (in zone Z2-La2) for gorse moor; 48.00513N, -2.231157W (in zone Z2-Bo4) for oak forest; and 48.00401N, -2.231281W (in zone Z2-Pa1) for meadow. For location of the zones, see Fig. 1. VOC – volatile organic compounds.

**Site**	**Gorse moor**		**Oak forest**		**Meadow**	
**Time frame**	day	night	day	night	day	night
**CO_2_ flux (mg.m^-2^.h^-1^)**	66.4 ± 0.2	65.1 ± 0.0	65.8 ± 1.5	65.5 ± 1.1	68.6 ± 4.8	71.1 ± 6.0
**VOC (µg.h^-1^.m^-2^)**	11.84 ± 5.04	10.97 ± 1.93	9.11 ± 1.71	4.63 ± 0.78	14.99 ± 8.18	9.54 ± 2.28
**Humidity (%)**	2.5		3.1		2.0	
**Organic Matter (%)**	8.4		9.3		6.0	
**Total Nitrogen (g.kg^-1^)**	4.0		3.3		3.1	
**Cation exchange capacity (meq.100g^-1^)**	11.6		3.1		9.8	
**Slaking index**	0.6		0.7		1.1	
**pH**	4.0		4.2		4.7	
**Texture**	Sandy-loam		Loam		Loam	
***Granulometry***:						
- **Clay (%)**	11.5		15.2		12.5	
- **Fine silt (%)**	18.6		26.8		24.1	
- **Coarse silt (%)**	18.3		23.6		27.7	
- **Fine sand (%)**	25.1		17.0		10.9	
- **Coarse sand (%)**	26.4		17.5		24.9	

**Table 3.** Climate parameters measured during the BioBlitz in 2017 in the study area of the Biological Field Station of Paimpont, University Rennes 1. The exact location of the weather station is 48.004373N, -2.228318W (in zone Z3_Pa2). For location of the zone, see Fig. 1.

**Time**	**18 July, 2 pm**	**18 July, 5 pm**	**19 July, 8 am**
Air pressure (in Hg)	29.83	29.71	29.69
Cloud base (ft)	1195.25	3108.85	1057.83
Evapotranspiration (mm.day^-1^)	0.056	0.076	0.005
Rainfall (mm.day^-1^)	0.22	0.22	14.9
Dewpoint (F)	60.81	66.09	63.01
Apparent temperature (F)	63.44	81.02	67.59
Temperature (F)	63.8	77.5	65.4
Humidity (%)	90	68	92
Solar radiation (W.m^2^)	46	663	47
Wind direction (°)	301	73	227
Wind speed (mph)	7	4	4
Wind gust direction (°)	306	42	230
Wind gust (mph)	18	17	12

**Table 4.** Physico-chemical parameters measured during the BioBlitz in 2017 in the water at different sites of the hydrological system in the study area of the Biological Field Station of Paimpont, University Rennes 1. The exact locations were 48.008425N, -2.2265516W (in zone Z1_Ea) for Etang d’en haut; 48.0079626N, -2.2268825W (in zone Z1_Fo) for Ruisseau de la grève 1; 48.0039444N, -2.230085W (in zone Z2_Ea) for Etang du Châtenay; 48.0032027N, -2.2302007W (in the zone Z2_Ea) for Dyke; and 48.0029530N, -2.2302378W (in the zone Z3_Bo1) for Ruisseau de la grève 2. The sites in the table are listed downstream. For location of the zones see Fig. 1. Temp – temperature, Cond – conductivity.The depth of measurement is indicated relative to the water surface.

**Site**	**Depth (m)**	**Temp (°C)**	**Cond (µS.cm^-1^)**	**O_2_ (%)**	**O_2_ (mg.l^-1^)**	**pH**
Etang d'en haut	0.0-0.1	22.61 ± 0.39	88.57 ± 1.33	87.16 ± 8.24	7.51 ± 0.71	6.70 ± 0.19
	0.1-0.5	23.04 ± 0.16	87.46 ± 0.44	89.70 ± 5.86	7.66 ± 0.48	6.66 ± 0.15
	0.5-1.0	22.09 ± 0.46	88.08 ± 0.49	96.2 ± 3.490	8.37 ± 0.29	6.69 ± 0.19
	1.0-1.5	20.02 ± 0.50	92.1 ± 3.549	97.3 ± 4.030	8.82 ± 0.36	6.58 ± 0.15
	1.5-1.8	19.51 0.75	97.23 ± 9.42	93.17 ± 6.82	8.52 ± 0.56	6.53 ± 0.14
Ruisseau de la grève 1	0.05	16.90 ± 0.01	119.61 ± 0.57	0.00 ± 0.96	0.00 ± 0.90	6.50 ± 0.02
Etang du Châtenay	0.0-0.1	24.88 ± 0.60	101.65 ± 1.36	89.89 ± 6.31	7.41 ± 0.51	6.79 ± 0.08
	0.1-0.5	25.27 ± 0.25	100.98 ± 0.95	92.31 ± 6.01	7.56 ± 0.47	6.79 ± 0.08
	0.5-1.0	24.88 ± 0.36	100.55 ± 0.96	92.81 ± 6.70	7.66 ± 0.53	6.75 ± 0.12
	1.0-1.5	22.58 ± 1.11	102.70 ± 2.97	98.41 ± 4.47	8.49 ± 0.48	6.77 ± 0.10
	1.5-1.8	20.79 ± 0.26	101.54 ± 1.27	97.89 ± 5.52	8.74 ± 0.46	6.70 ± 0.14
	1.8-2.5	19.64 ± 0.58	105.51 ± 5.04	92.95 ± 7.21	8.48 ± 0.58	6.59 ± 0.16
	2.5-3.0	17.16 ± 0.39	126.68 ± 6.13	70.43 ± 6.77	6.76 ± 0.62	6.33 ± 0.03
Dyke	0.88	24.96 ± 0.13	99.24 ± 0.16	85.77 ± 2.47	7.07 ± 0.19	6.67 ± 0.04
Ruisseau de la grève 2	0.00	19.19 ± 0.04	104.00 ± 0.00	91.37 ± 1.36	8.44 ± 0.13	6.49 ± 0.02

**Table 5.** Physico-chemical parameters measured during the BioBlitz in 2017 in the water at different sites of the hydrological system in the study area of the Biological Field Station of Paimpont, University Rennes 1. The exact locations were 48.008425N, -2.2265516W (in zone Z1_Ea) for Etang d’en haut; 48.0079626N, -2.2268825W (in zone Z1_Fo) for Ruisseau de la grève 1; 48.0039444N, -2.230085W (in zone Z2_Ea) for Etang du Châtenay; 48.0032027N, -2.2302007W (in the zone Z2_Ea) for Dyke; and 48.0029530N, -2.2302378W (in the zone Z3_Bo1) for RG2 - Ruisseau de la grève 2. The sites in the table are listed downstream. For location of the zones, see Fig. 1. Chl a – Chlorophyll "a", Phyco – Phycocyanin, Turb – turbidity, PAR – photosynthetically active radiation. The depth of measurement is indicated relative to the water surface.

**Site**	**Depth (m)**	**Chl a (RFU)**	**Phyco (RFU)**	**Turb (NTU)**	**PAR (μE.m^−2^.s^−1^)**
Etang d'en haut	0.0-0.1	2.40 ± 2.80	7.23 ± 3.69	14.18 ± 12.71	569.24 ± 183.25
	0.1-0.5	34.11 ± 7.38	11.86 ± 1.70	1.88 ± 0.88	188.07 ± 75.81
	0.5-1.0	52.15 ± 8.40	15.89 ± 2.09	1.53 ± 0.21	53.08 ± 15.75
	1.0-1.5	54.39 ± 17.35	11.38 ± 3.16	1.72 ± 0.43	25.75 ± 3.47
	1.5-1.8	48.12 ± 17.14	10.83 ± 3.92	1.80 ± 0.48	
Ruisseau de la grève 1	0.05	1.45 ± 0.24			
Etang du Châtenay	0.0-0.1	5.28 ± 2.11	9.80 ± 7.72	7.02 ± 4.02	1241.65 ± 548.96
	0.1-0.5	12.26 ± 3.84	7.54 ± 1.79	2.53 ± 1.00	402.78 ± 164.55
	0.5-1.0	15.06 ± 4.57	8.77 ± 1.72	2.85 ± 0.42	128.17 ± 73.76
	1.0-1.5	19.81 ± 7.85	16.73 ± 10.71	3.90 ± 1.28	56.82 ± 19.67
	1.5-1.8	44.03 ± 17.72	16.57 ± 5.14	3.90 ± 0.54	23.22 ± 4.50
	1.8-2.5	14.36 ± 6.31	8.84 ± 1.65	5.20 ± 2.15	8.80 ± 3.06
	2.5-3.0	12.47 ± 2.22	7.41 ± 1.94	18.31 ± 4.40	2.51 ± 0.37
Dyke	0.88	17.40 ± 1.21	10.04 ± 0.86	2.68 ± 0.53	404.84 ± 28.91
Ruisseau de la grève 2	0.00	0.22 ± 0.17	0.16 ± 0.05	8.74 ± 7.87	

**Table 6.** Physico-chemical parameters measured during the BioBlitz in 2017 in the water at different sites of the hydrological system in the study area of the Biological Field Station of Paimpont, University Rennes 1. The exact locations were 48.008425N, -2.2265516W (in zone Z1_Ea) for Etang d’en haut; 48.0079626N, -2.2268825W (in zone Z1_Fo) for Ruisseau de la grève 1; 48.0039444N, -2.230085W (in zone Z2_Ea) forEtang du Châtenay; and 48.0029530N, -2.2302378W (in the zone Z3_Bo1) for Ruisseau de la grève 2. The sites in the table are listed downstream. For location of the zones, see Fig. 1. TP – total phosphorus, TN – total nitrogen, DOC - dissolved organic carbon, TDS – total dissolved solids, ORP – oxidoreduction.

	**Etang d'en haut**	**Etang du Châtenay**	**Ruisseau de la grève 1**	**Ruisseau de la grève 2**
P-PO_4_ (mg.l^-1^)	<0.011	<0.011		
N-NO_3_ (mg.l^-1^)	<0.15	<0.15		
TP (mg.l^-1^)	<0.019	<0.019		
TN (mg.l^-1^)	0.76	0.69		
DOC (mg.l^-1^)	10.43	8.78		
TDS (g.l^-1^)			0.08 ± 0.00	0.07 ± 0.00
ORP (mV)			224.42 ± 1.40	233.06 ± 0.33
