# Supplementary figures and images for: Transdisciplinary Bioblitz: Rapid biotic and abiotic inventory allows studying environmental changes over 60 years at the Biological Field Station of Paimpont (Brittany, France) and opens new interdisciplinary research opportunities
Source: Biodivers Data J. 2020 Mar 27;8:e50451. doi: 10.3897/BDJ.8.e50451 (PMC7125239; doi:10.3897/BDJ.8.e50451)

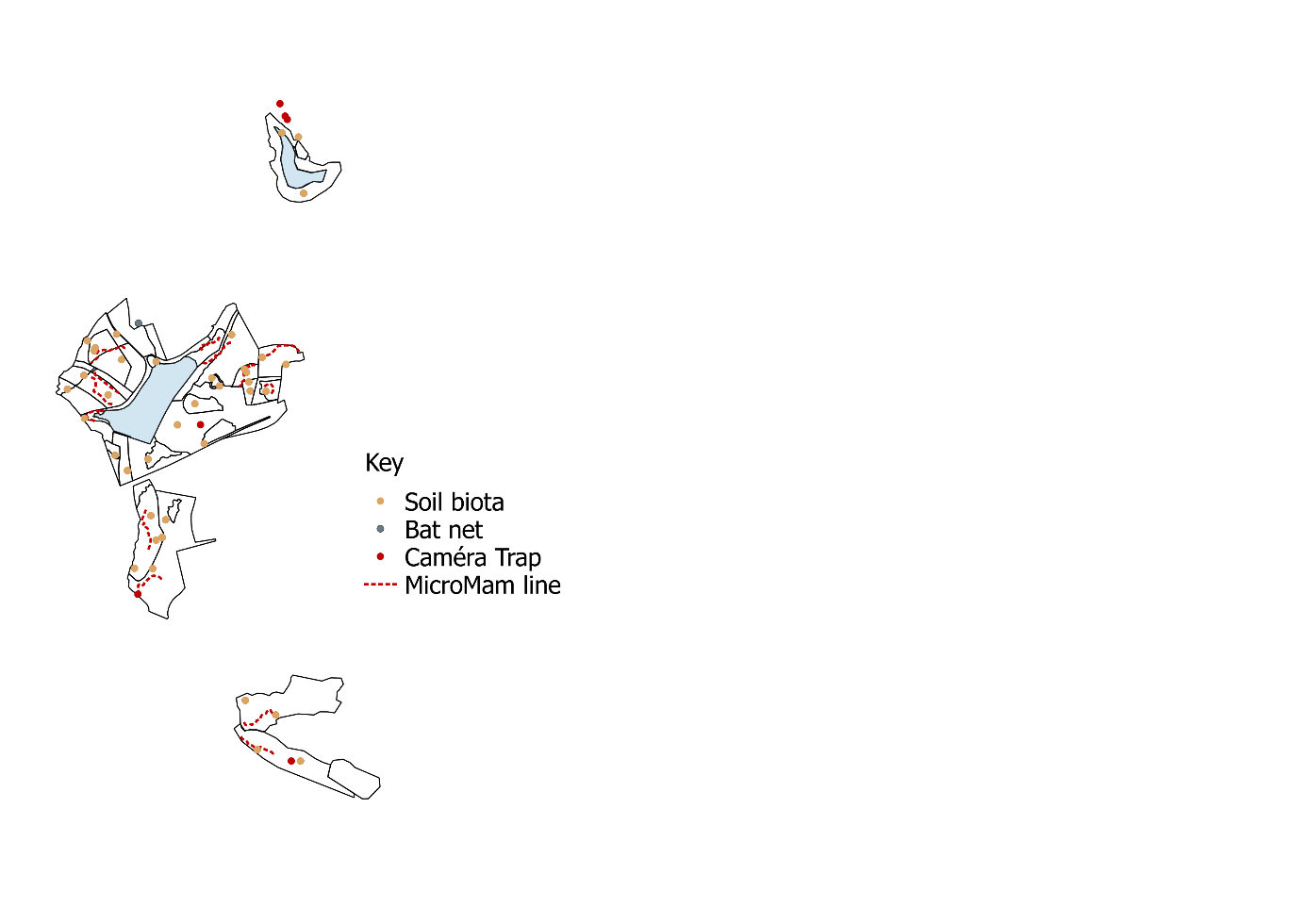

Supplement: Supplementary material 3 — Sampling points [file bdj-08-e50451-s003.png]

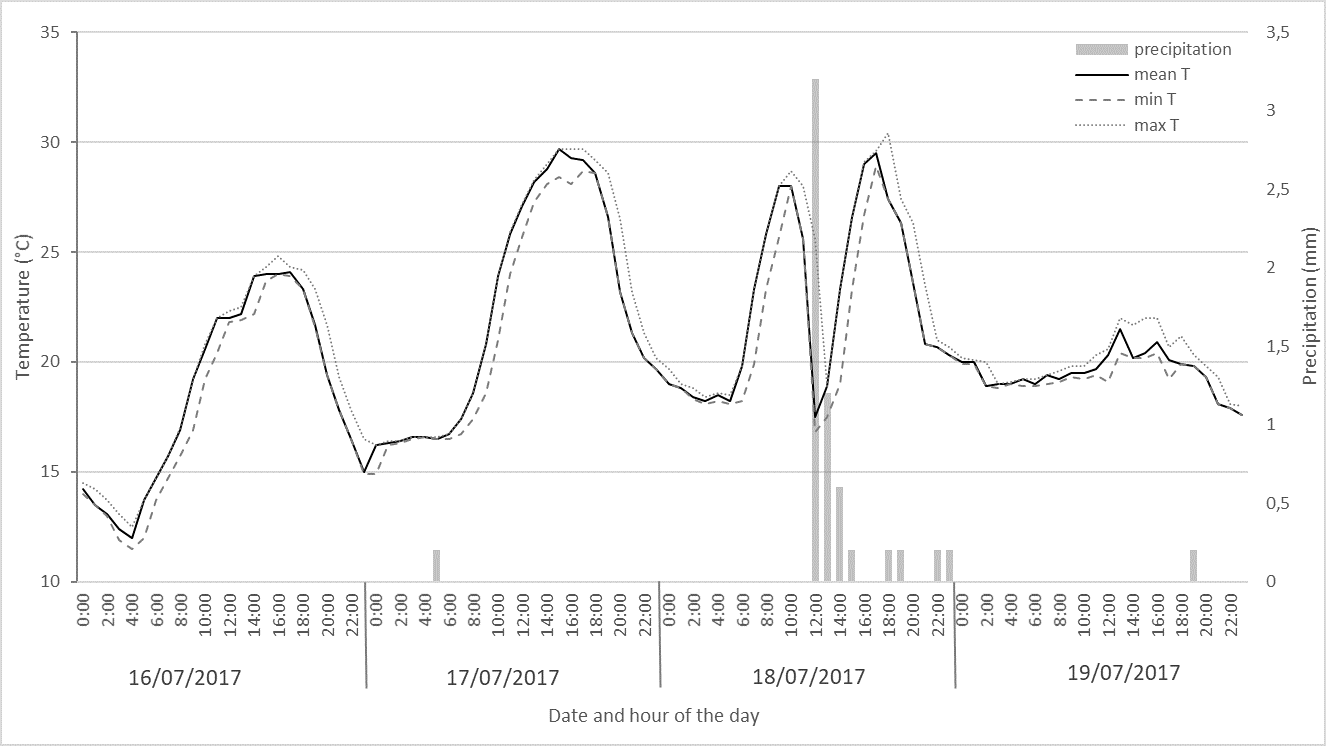

Supplement: Supplementary material 4 — Climatic conditions during BioBlitz [file bdj-08-e50451-s004.png]

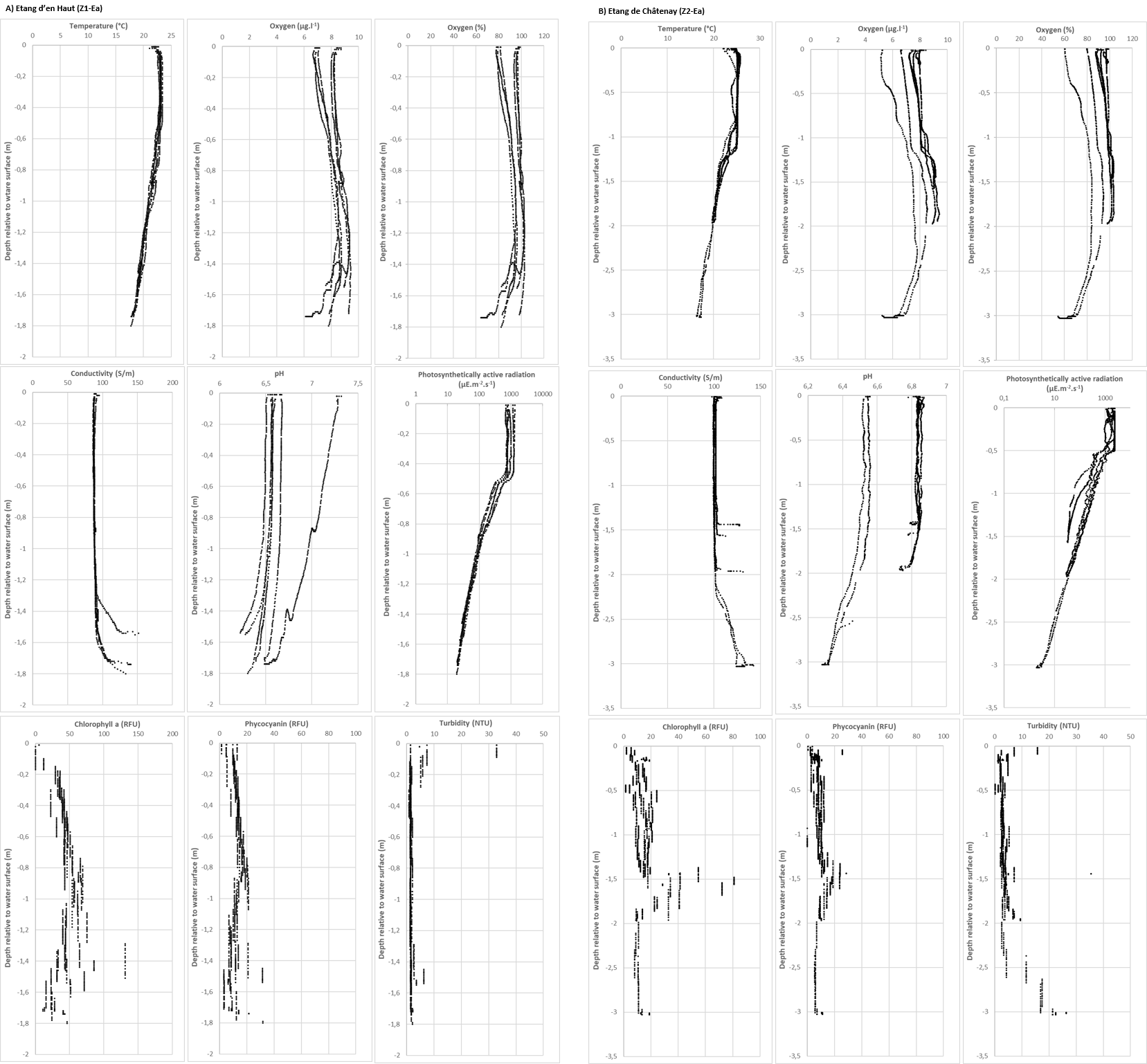

Supplement: Supplementary material 5 — Profiles of abiotic water parameters [file bdj-08-e50451-s005.png]
